# Technical efficiency among agricultural households and determinants of food security in East Java, Indonesia

**DOI:** 10.1038/s41598-021-83670-7

**Published:** 2021-02-18

**Authors:** Rachman Hakim, Tri Haryanto, Dyah Wulan Sari

**Affiliations:** 1 Department of Economics, Madura University, Panglegur Street Km 3.5, Pamekasan, 69317 Indonesia; 2 Department of Economics, Airlangga University, Surabaya, Indonesia

**Keywords:** Environmental economics, Sustainability

## Abstract

Rice is a staple food in East Java, and the average consumption is 100 kg/capita/year. However, rice productivity has declined dramatically in recent years. Food security can be reached by improving the technical efficiency of rice farming, especially in rice farming centers such as East Java Province. This study aims to measure technical efficiency and its determinants using two limit tobit. And it also aims to examine the effect of the technical efficiency of rice farming on food security using logit regression. Technical efficiency will be measured by using data envelopment analysis (DEA). The results show that the technical efficiency of rice farming is very low in East Java. Government assistance, irrigation, and extension have a significant effect on technical efficiency. Meanwhile, membership of farmer organization has no effect on technical efficiency. Around 69% of farmers can be categorized as food secure households. The estimation of logit regression shows that household size, income, land size, education, age, and gender significantly influence food security in East Java. Meanwhile, credit and technical efficiency did not have any significant effect.

## Introduction

Agriculture is a dominant sector in Indonesia, including in East Java Province. East Java Province was the largest producer of rice during 2013–2017^1^. The lowest layer of Indonesia's population pyramid is farmers and fishermen who live in rural areas. There are a lot of people working in the agricultural sector. The data shows that the population of East Java aged 15 years and over amounted 6,054,066 people are more likely to work in the agricultural sector (crops, horticulture, plantations, and livestock) which are divided into 3,591,231 men and 2,462,835 women. Meanwhile, 13,519,716 people work in the non-agricultural sector. East Java is the province with the largest population working in the agricultural sector compared to other provinces in Indonesia^2^. It indicates the importance of the agricultural sector in East Java.

The agriculture sector is divided into food crops, horticulture, plantation, livestock, forestry, and fisheries sub-sectors. However, this research focuses on investigating food crops. Food crops are very identical to rice/rice plants because most of the population of Indonesia (including East Java) consume rice as a staple food. It is supported by the data from the Ministry of Agriculture which shows that the average consumption of rice is 100 kg/capita/year. Meanwhile, the consumption of tubers, beans, fruit, or meat per capita is less than 10 kg/year^1^.

A large number of rice needs should be followed by the production of rice plants that meet these needs. However, the productivity of rice plants must be increased so that the needs of rice plants or food security are maintained.

**Figure 1:**
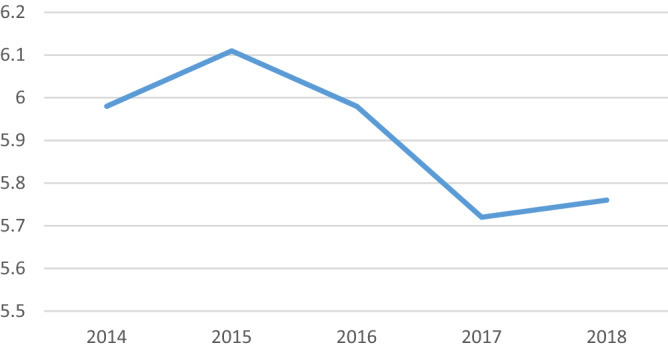
East Java rice crop productivity in 2014–2018 (Ton/Ha). Source: Central Bureau of Statistics.

Figure 1 shows that rice crop productivity in East Java has declined in the last five years (2014–2018), especially since 2016. This situation caused East Java Province had to import 96.51 million USD worth of rice from Thailand had in 2018^3^. Declining rice crop productivity can be a signal to take corrective steps so that rice production can meet the needs of the people of East Java.Technical efficiency has an important role in increasing rice productivity.

Technical efficiency is influenced by several factors. Haryanto et al. examined the effect of several factors on the efficiency of rice farmers in several regions in Indonesia, including the factor of government assistance and irrigation. The results show that the government assistance has an effect on the efficiency of rice farming in all areas studied even in Indonesia as a whole^4^.

Karunarathna and Wilson stated that inefficiency is strongly influenced by membership in farmer organizations. Membership in farmer organizations is important because farmers can obtain various facilities such as training, cheap fertilizers, and others^5^. Islam et al.^6^ and San et al.^7^ also stated that extension is very important to increase technical efficiency.

The agricultural sector is certainly much more related to food security compared to other sectors^8^. Several studies related to the efficiency of agricultural production and food security have been conducted several times. Ogundari in his research suggested that efforts to strengthen food security and poverty alleviation can be done by increasing the efficiency of agriculture and food production^9^. Koirala et al. also stated the same thing^10^. Food security is a major problem in the Philippines. In addition, food security is greatly influenced by farmers' production decisions, land reform programs, and technical inefficiencies in rice production.

Adeniyi and Dinbabo examined the relationship between technical efficiency and food security using the multinomial regression method. Technical efficiency with SFA showed that the average efficiency was 0.85. It also found that there is a positive and significant relationship between technical efficiency, income and food security^11^. Majumder et al. in their research stated that there is a way to reduce post-harvest losses of rice and increasing food security in Bangladesh. Improving the technical efficiency of the rice production system in Bangladesh can improve food security^12^. Iheke and Onyendi measured technical efficiency and food security using the SFA method and food security index based on daily food intake in Nigeria. The conclusion from this study is: food insecurity is prevalent among agricultural households and food security is very difficult to achieve by farm households even if it is efficient in agricultural management^13^.

Oyakhilomen et al. examined the relationship between technical efficiency and food security of chicken egg-farmers in Nigeria. The method used is SFA, a measure of food security based on the US Department of Agriculture (USDA) and correlation analysis. The results showed that the level of technical efficiency was very low, 23% and 90% of farmers were categorized as food vulnerable. Based on the correlation analysis, it was found that there was a positive relationship between technical efficiency and food security^14^. Oyetunde-Usman and Olagunju examined the relationship between technical efficiency and household food security of farmers using the SFA method and the probit model. The results showed that food-resistant farming households were more technically efficient than food-vulnerable households^15^. Basically, food security is not only influenced by technical efficiency. There are many more factors that affect food security.

In addition to the efficiency of rice production, there are still many other factors that affect food security. The research conducted by Abdullah et al. revealed that gender has a significant effect on food security^16^. Female household heads tend to be vulnerable in maintaining family food security. Agriculture is a sector that is very identical to men's work because it drains a lot of energy and thoughts so it is not suitable for women. Holden and Ghebru in their research stated that climate change significantly affects agricultural productivity so that it will ultimately have an impact on food security^17^.

Mishra et al. argued that increasing rice crop production and identifying the causes of inefficiency in rice crop production can help farmers in obtaining higher incomes, increasing food security, and alleviating poverty. Furthermore, research is needed to find out what factors influence inefficiencies in rice crop production so that food security will be maintained^18^.

Emran and Shilpi in their study revealed that labor wages affect poverty alleviation and food security in rural areas^19^. Mellor and Malik conducted a study in Pakistan and found that the growth of the agricultural sector had a dominant effect on poverty alleviation and food security^20^. Montaud et al. conducted a study related to agricultural yields and poverty in Nigeria using the Computable General Equilibrium (CGE) method. The results of his research suggested that a long-term decline in agricultural output in Nigeria results in poverty^21^.

Several factors are considered to affect household food security including household assets^22^; home ownership^23^; household savings^24^; financial limitations^25^; education^26^; livestock ownership^27^; unemployment and income^28^; knowledge about food storage, processing, and nutrition^29^; corruption, fiscal errors, large debts, and inconsistent government policies^30^; off-farm job^31^; gender^32^; family size, land area, soil fertility, access to irrigation, fertilizer use, seed utilization^33^; delivery and access to market information, age^34^; dependency ratio, electricity connection, irrigation availability^35^; monthly income, family structure^36^; and the existence of infrastructure^37^. There are many factors that affect food security. However, each study adjusts to the availability of data in the field when the research was conducted.

Therefore, to improve the structure of the national economy, it is necessary to make improvements in the agricultural sector. The agricultural sector is closely related to food security issues. The efficiency of rice production is certainly expected to be a solution in the process of increasing food security in East Java, Indonesia. This is the basis of interest to examine the relationship between technical efficiency and food security in East Java, Indonesia.

## Material and methods

### Area descriptions

The data used in this research is secondary data obtained from the Central Bureau of Statistics in the form of Agriculture Survey (Agriculture Household Income Survey) data for the East Java Province in 2013.The samples were 8603 farm households.

### Data envelopment analysis

To measure the efficiency of rice production, the Data Envelopment Analysis (DEA) method was used. Data Envelopment Analysis (DEA) is a method of optimizing a mathematical program that measures the technical efficiency of a Decision Making Unit (DMU) and compares it relative to other DMU that use the same type of input and output. DEA formulates DMU as a fractional linear program to find a solution if the model is transformed into a linear program with the weights of input and output. The relative efficiency of DMU in DEA is also defined as the ratio of total weighted output divided by total weighted output (total weighted output).

DEA assumes that each DMU will have a weight that maximizes its efficiency ratio (maximizing total weighted output/total weighted input). The assumption of maximizing the efficiency ratio uses output orientation in calculating technical efficiency. Another orientation is to minimize input, but both assumptions will get the same results.

A DMU is relatively efficient if the dual value is equal to 1 (100 percent efficiency). If the dual value is less than 1, the DMU is considered to be relatively inefficient. The DEA model is divided into two, namely Constant Return to Scale (CRS) and Variable Return to Scale (VRS).There are two orientations commonly used in the efficiency measurement method using DEA, input-oriented and output-oriented.

The efficiency of rice production is analyzed using the Output-Oriented Data Envelopment Analysis (DEA) method with the assumption of Variable Return to Scale (VRS). This efficiency will be estimated by DEAP 2.1.

$$ \begin{aligned} & {\text{Max}}_{{\theta_{i} ,\lambda_{i} }} \theta_{i} \\ & s.t. \theta_{i} y_{i} - Y\lambda_{i} \le 0 \\ & X\lambda_{i} - X_{i} \le 0 \\ & j^{\prime } \lambda = 1 \\ & \lambda_{i} \ge 0 \\ \end{aligned} $$

The output variable used is rice (IDR). These inputs consist of land area/harvest area (m2), seeds (IDR), fertilizer (IDR), and labor (IDR).

### Determinants of technical efficiency

Tobit regression is very adequate to represent the model of the efficiency effect. Two limit Tobit will be used because the value of rice production efficiency is in the range of 0 to 1. The general specification of the tobit model is as follows.

$$ \begin{aligned} TE_{I}^{*} & = \beta^{\prime } Z_{i} + \varepsilon_{i} \\ TE_{i} & = L_{1i} \quad if TE_{i}^{*} \le L_{1i} \\ & = TE_{I}^{*} \quad if L_{1i} < TE_{I}^{*} < L_{2i} \\ & = L_{2i} \quad if TE_{i}^{*} \le 1 \\ \end{aligned} $$

where $TE_{i}^{*}$ is a latent variable that represents an index of technical efficiency, $TE_{i}$ is the dependent variable being reviewed, $Z_{i}$ is an explanatory variable vector that represents the characteristics of agriculture, $\beta$ is a vector of parameters to be estimated, $u_{i}$ is *error term*, $L_{1i}$ and $L_{2i}$ are the lower and upper limit. For more details with the following specifications:

$$ TE_{i} = \delta_{0} + \delta_{1} Z_{1i} + \delta_{2} Z_{2i} + \delta_{3} Z_{3i} + \delta_{4} Z_{4i} + \varepsilon_{i} $$

where $TE_{i}$ is the technical efficiency of farmers in rice production; *Z*_*1i*_isgovernment assistance (D = 1 if farmers have accepted the government assistance, D = 0 otherwise), *Z*_2*i*_is dummy membership of a farmer organization (D = 1 if farmer is a member of farmer organization, D = 0 otherwise), *Z*_3*i*_is a dummy for irrigation (D = 1 for irrigated rice fields, D = 0 otherwise), and*Z*_4*i*_is agricultural extension (D = 1 if farmers have accepted agricultural extension, D = 0 otherwise). $\delta_{0}$ is the parameter to be estimated, $\varepsilon_{i}$ is a random variable that is assumed to be normally distributed. This model will be estimated by the maximum likelihood method with STATA.13.

### Food security

There are many definitions of food security. This term usually implies indirectly that people have the same and sustainable economic and physical access to adequate amounts of food nutrition to meet daily calorie needs and to maintain an active and healthy lifestyle. Complex definitions make food security is quite difficult to calculate. Food availability is the basis for food access and food security^38^, especially at the household level. One way to measure food security can use a simple method of measuring the ratio of household food availability as an indicator of food security based on the opinion of Frelat et al^39^.

Ridaura et al. measure household food security by looking at potential food availability^40^. This indicator of measurement of potential food availability (PFA) is measured based on the calorie of calories per farm household based on reports on production and consumption of agricultural products and supplies. Dithmer and Abdulai also measure food security based on food energy consumption as indicated by kilocalories (kcal) per day^41^. Muraoka says that the amount of food consumption can be used to describe food security^42^. Swindale and Bilinsky measure food security using the Household Dietary Diversity Score (HDDS) method^43^. To put it simply, farm households that have food security = 1. Whereas farm households that experience food vulnerability = 0.

To determine whether a farmer's household is food resistant or not, the following formula will be used in this research:

$$ {\text{Food}}\;{\text{Security}}\;{\text{Score}} = \frac{A + B + C + D}{4} $$

where A = 1 if have a food supply, otherwise A = 0; B = 1 if have enough food supply, otherwise B = 0, C = 1 if never have experienced food shortages, otherwise C = 0; D = 1 if there are no toddlers who have below normal body weight, otherwise D = 0.

Food security scores will range from 0–1. If the food security score = 1 then the farm household can be said to be food security (D = 1). If the food security score is < 1 then the farm household can be said to be food insecure/vulnerable to food (D = 0).

### Determinants of food security

The estimation of the food security model will use logit regression because the value of food security is between 0 and 1. The specifications of the food security model will be estimated as follows.

$$ L_{i} = ln\left( {\frac{{P_{i} }}{{1 - P_{i} }}} \right) = \alpha_{0} + \alpha_{1} Q_{1i} + \alpha_{2} Q_{2i} + \alpha_{3} Q_{3i} + \alpha_{4} Q_{4i} + \alpha_{5} Q_{5i} + \alpha_{6} Q_{6i} + \alpha_{7} Q_{7i} + \alpha_{8} Q_{8i} + \varepsilon_{i} $$

where $ln( {\frac{{P_{i} }}{{1 - P_{i} }}})$ is odds ratio for the occurrence of value 1 (food security), Q are factors that explain food security. The factors are Q_1_ = household size (in person), Q_2_ = income (in log), Q_3_ = land size (in log), Q_4_ = credit (D = 1 for farmers who have access credit, otherwise D = 0), Q_5_ = education (D = 1 for high school education and tertiary education level, otherwise D = 0), Q_6_ = age (in year), Q_7_ = gender (D = 1 for male, D = 0 for female) and Q_8_ = technical efficiency (0—1). α_1_, α_2_, …, α_7_ are the parameters to be estimated, ε_i_ is a random variable that is assumed to be normally distributed. This model will be estimated using the Maximum Likelihood Estimation (MLE) method with STATA.13.

## Results and discussion

Data Envelopment Analysis estimation results show that the technical efficiency of rice farming is very low in East Java, which is in average of 0.27.

Table 1 shows that most farmers have technical efficiency in the range of 0.101–0.200 and 0.201–0.300 (2735 and 2745 farmer households). Meanwhile, farmers who have very little technical efficiency are in the range of 0.701–0.800, 0.801–0.900, and 0.901–1000 (62, 37, and 75 farmer households). Many things need to be done to improve this technical efficiency. This should receive more attention from the East Java Government because basically technical efficiency is one of the keys to increase rice productivity.

**Table 1:** Distribution of technical efficiency.

	DMU	%
0.000–0.100	457	5.31
0.101–0.200	2735	31.79
0.201–0.300	2745	31.91
0.301–0.400	1486	17.27
0.401–0.500	632	7.35
0.501–0.600	264	3.07
0.601–0.700	110	1.28
0.701–0.800	62	0.72
0.801–0.900	37	0.43
0.901–1.000	75	0.87
Total	8603	100

Returns to scale distribution of rice farming in East Java is presented in Table 2. The efficiency scale of most rice farmers in East Java shows decreasing returns to scale (DRS), amounting to 6323 farmer households. Meanwhile, there are increasing returns to scale (IRS) of 1,847 farmer households and constant returns to scale (CRS) of 433 farmer households. It can be concluded that most of the efficiency scale of farmer households obtains the decreasing returns to scale (DRS), which is around 73.50%. Meanwhile, the IRS is around 21.47% and the CRS is around 5.03%.

**Table 2:** Distribution of returns to scale.

	DMU	%
CRS	433	5.03
DRS	6323	73.50
IRS	1847	21.47
Total	8603	100

Table 3 shows the comparison between food secure and non-food secure households. Farmers with food secure category around 69% or 5916 farmers, 31% or 2,687 farmers have a risk of food vulnerability (non-food secure). Household size of rice farmers ranges from 1–16 people and the mean difference between food secure and non-food secure households is 0.05. The mean income is 6,500.81 thousand rupiahs for food secure households and 3371.47 thousand rupiahs for non-food secure households. Land size also has a very striking difference, the mean difference is 2168.17 m2. Farmers have very minimal access to credit, only 8% of farmers can access credit. Only 12% of the food secure households are highly educated, while only 7% of the non-food secure households are highly educated. The average age of farmers reaches 52 years which can be indicated that most farmers in East Java are categorized as experienced farmers. Gender of farmers is dominated by men, which is around 88%. This is because the agricultural sector is indeed very identical to men's work. Meanwhile, female farmers are only 12%. About 40% of farmers receive government assistance. 42% of food secure households and 30% of non-food secure households join the farmer organization. 53% of food secure households and 37% of non-food secure households get rice field irrigation. 26% of food secure households and 16% of non-food secure households get agricultural extension.

**Table 3:** Descriptive statistics.

Variable	Food secure				Non-food secure				Mean difference
	n = 5916				n = 2687				
	Min	Max	Mean	STD	Min	Max	Mean	STD	
Household size	1	13	3.73	1.56	1	16	3.78	1.62	0.05
Income	1	902,852	6500.81	17,045.73	3	140,003	3371.47	5205.69	3129.34
Land size	50	250,000	5416.25	8890.38	50	90,800	3248.08	4495.24	2168.17
Credit	0	1	0.09	0.28	0	1	0.07	0.25	0.02
Education	0	1	0.12	0.32	0	1	0.07	0.25	0.05
Age	16	98	52.59	12.58	20	97	51.16	12.72	1.43
Gender	0	1	0.89	0.32	0	1	0.85	0.35	0.04
Technical efficiency	0.017	1	0.27	0.15	0.017	1	0.26	0.14	0.01
Gov. assistance	0	1	0.40	0.49	0	1	0.43	0.50	0.03
Membership	0	1	0.42	0.49	0	1	0.30	0.46	0.12
Irrigation	0	1	0.53	0.50	0	1	0.37	0.48	0.16
Extension	0	1	0.26	0.44	0	1	0.16	0.37	0.10

The estimation result for tobit regression is presented in Table 4. Government assistance, irrigation, and extension have a significant effect on technical efficiency. Meanwhile, membership of farmer organization has no effect on technical efficiency.

**Table 4:** Estimation for Tobit model.

Variable	Coefficient	Stand. error	t	*P*
Constant	0.26	0.00	96.13	0.00***
Gov. assistance	− 0.00	0.00	− 1.95	0.05**
Membership of farmer organization	0.01	0.00	0.45	0.66
Irrigation	0.01	0.00	3.86	0.00***
Extension	0.01	0.00	2.94	0.00***
Log likelihood	4078.65			
LR test	36.23***			

***; **; * indicate significance at 1%, 5% and 10% level.

Government assistance has a negative and significant effect. If farmers receive government assistance, it means that technical efficiency will decrease. This can be due to farmers' lack of knowledge due to low levels of education. Irrigation has a positive and significant effect. Irrigation is very important in rice farming. If the rice fields are adequately irrigated, then the rice can develop well. Extension has a positive and significant effect.

The estimation result for logit regression is presented in Table 5. The estimation shows that most of the variables have a significant effect on food security in East Java, except for credit variables which have no significant effect.

**Table 5:** Estimation for Logit regression.

Variable	Coefficient	Odds ratio	Std. error	Z	*P* value
Constant	− 3.34	0.04	0.01	− 14.17	0.00***
Household size	− 0.03	0.97	0.01	− 2.05	0.04**
Income	0.55	1.74	0.13	7.57	0.00***
Land size	0.46	1.59	0.14	5.22	0.00***
Credit	0.09	1.09	0.10	0.97	0.33
Education	0.49	1.64	0.15	5.37	0.00***
Age	0.01	1.01	0.00	4.96	0.00***
Gender	0.18	1.19	0.09	2.44	0.01**
Technical efficiency	0.24	1.27	0.21	1.41	0.16
Log likelihood					− 5104.16
LR test					475.82***

***; **; * indicate significance at 1%, 5% and 10% level.

Household size has a negative and significant effect with odds ratio = 0.97 and *p* = 0.04. This means that more members in the family will increase the likelihood that the family experience food vulnerability. Income has a positive and significant effect with odds ratio = 1.74 and *p* = 0.00. If the income of farmers increases, it will increase their food security. Land size has a positive and significant effect with odds ratio = 1.59 and *p* = 0.00. It means that if the size of land owned is larger, farmers will increase food security. Education is one of the most influential variables on food security in East Java. Education has a positive and significant effect with odds ratio = 1.64 and *p* = 0.00. It means that if the farmers have a higher level of education, the farmers will increase food security. Age has a positive and significant effect with odds ratio = 1.01 and *p* = 0.00. It means that farmers who have more experiences (indicated by age) will increase their food security. Gender has a positive and significant effect with odds ratio = 1.19 and *p* = 0.01. It means that male farmers will increase the likelihood that the family experience food security. Meanwhile, access to credit has no significant effect on food security.

The result of research on the effect of technical efficiency is quite surprising. Technical efficiency has no significant effect on food security in East Java. The results of this study are different from the results of research by Ogundari and Koirala et al. which stated that the technical efficiency of the agricultural sector influenced food security. The results of this study are also different from those of Ogundari^9^, Koirala et al.^10^, Adeniyi and Dinbabo^11^, Majumder et al.^12^, Oyakhilomen et al.^13^, as well as Oyetunde-Usman and Olagunju^14^ which stated that the technical efficiency of the agricultural sector affected food security. Meanwhile, the results of research by Iheke and Onyendi are the same with the results of this study which revealed that technical efficiency does not have a significant effect on food security^15^.

Data shows that the level of education of farmers in East Java is relatively low. Farmers who passed primary education were 5,414,605 people (84.34%), secondary education were 575,974 people (14.11%), and higher education 63,487 people (1.51%). This is quite alarming because education has a significant effect on food security in East Java. Based on age, farmers aged 15–24 years were 366,145 people (6.05%), 25–59 years were 4,068,510 people (67.20%), and > 60 years were 1,619,411 people (26.75%). It indicates that most farmers have reached a mature age. But the number of farmers aged 15–24 years is enough to attract attention because it is a sign that only a few young people want to work in the agricultural sector in East Java. Problems with education and age should be resolved if the East Java Provincial Government is able to convince the community that agriculture is a viable and profitable source of livelihood. So that people who are young and highly educated will interest to work in the agricultural sector. If this cannot be done, it is not surprising that more people choose to work in the non-agricultural sector.

The low level of technical efficiency of rice farming can be caused by a lack of community knowledge to manage agriculture well. One solution from the government is an agricultural extension. Growth in the number of agricultural extension workers in East Java in 2019 increased by 6.58% compared to the previous period. This is one of the East Java Provincial Government's commitments to increase public knowledge about a good agricultural system so that farmers’ hope to become more technically efficient and ultimately increase food security.The fact is that technical efficiency does not have a significant effect on food security. This can be due to very low technical efficiency. In addition, agriculture is no longer the main occupation for some farmers. Farmers usually have other income as the main source to support the household. So it is not surprising that income is more influential than technical efficiency.

**Figure 2:**
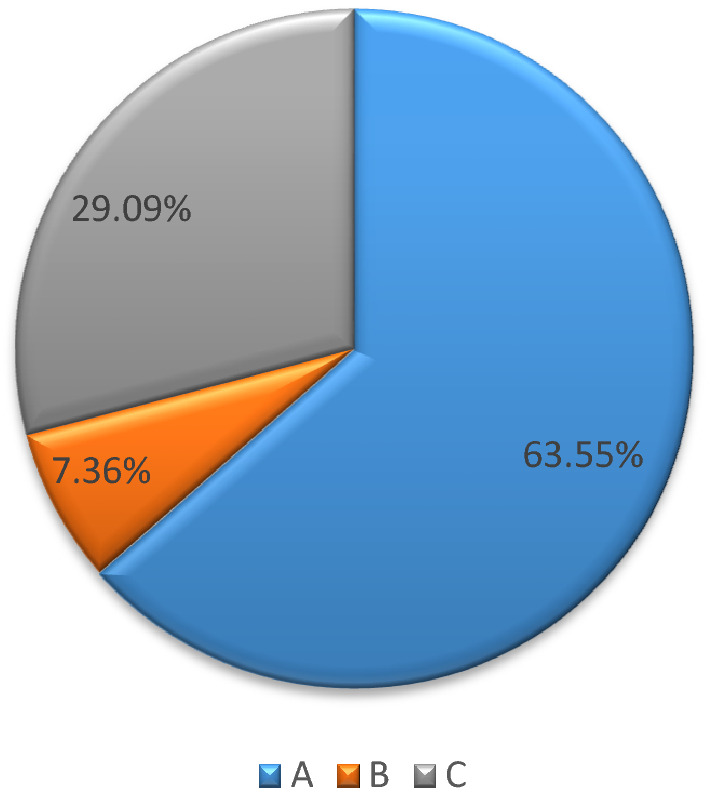
East Java farmer category.

Figure 2 shows that rice farmers are divided into several categories: (A) main income from rice farming activities; (B) main income from rice farming activities but have additional income; (C) main income from other activities instead of rice farming activities.

Based on the research sample, there are 63.55% of farmers who have their main income from rice farming activities. 7.36% of farmers have their main income from rice farming activities but still have additional income. In addition, 29.09% of farmers have their main income from other activities instead of rice farming activities.

## Conclusion

The technical efficiency of rice farming is very low in East Java. Even though East Java is one of the centers of rice farming. Government assistance, irrigation, and extension have a significant effect on technical efficiency. Meanwhile, membership of farmer organization has no effect on technical efficiency.

About 69% of farmers are included in the food secure category, while 31% of farmers are at risk of food vulnerability. The estimation of logit regression shows that household size, income, land size, education, age, and gender significantly influence food security in East Java. Meanwhile, credit and technical efficiency did not have a significant effect.Technical efficiency does not have a significant effect on food security, the government must continue to strive for improving this technical efficiency. The income generated in the agricultural sector also affects food security. The income is related or determined by the productivity of rice farming. The level of productivity must be maintained because if rice productivity decreases, it can certainly reduce the income and food security of farmers in East Java.

Farmer’s education is relatively low in East Java. Educated farmers are an important factor because they are expected to be more open-minded about innovations in agriculture to increase technical efficiency. Increasing the efficiency of rice farming can be done by increasing the interest of the educated population to take part in the rice farming management process due to the fact that most of the rice farmers in East Java are still low educated, with the average age is over 50 years old. Most of the farmers are 50 years old and only a small portion of 15–24 years old people work as farmers. In addition, agriculture is no longer the main occupation. Farmers usually have other income as the main source to support the household.

## References

[1] Ministry of Agriculture Republic of Indonesia. Agricultural Statistics 2018. 101 (Center for Agricultural Data and Information System, 2018).

[2] Ministry of Agriculture Republic of Indonesia. Statistik Ketenagakerjaan Sektor Pertanian Agustus 2019. 6–13 (Center for Agricultural Data and Information System, 2019).

[3] Central Bureau of Statistics. Statistik Impor Provinsi Jawa Timur 2018. Preprint at https://jatim.bps.go.id/publication/download.html?nrbvfeve=YzgyMWQ1NzgyNjAwMzcyYTIzNzdkNzkx&xzmn=aHR0cHM6Ly9qYXRpbS5icHMuZ28uaWQvcHVibGljYXRpb24vMjAxOS8wOS8yMC9jODIxZDU3ODI2MDAzNzJhMjM3N2Q3OTEvc3RhdGlzdGlrLWltcG9yLXByb3ZpbnNpLWphd2EtdGltdXItMjAxOC5odG1s&twoadfnoarfeauf=MjAyMC0xMC0yMCAyMDo1Mzo0NA%3D%3D (2019).

[4] Haryanto T, Talib BA, Salleh NHM (2015). An Analysis of technical efficiency variation in indonesian rice farming. J. Agric. Sci..

[5] Karunarathna M, Wilson C (2017). Agricultural biodiversity and farm level technical efficiency: an empirical investigation. J. For. Econ..

[6] Islam KMZ, Backman S, Sumelius J (2011). Technical, economic and allocative efficiency of microfinance borrowers and non-borrowers: evidence from peasant farming in Bangladesh. Eur. J. Soc. Sci..

[7] San NW, Abdlatif I, Mohamed ZA (2013). Farm efficiency and socioeconomic determinants of rain-fed rice production in Myanmar: a non-parametric approach. Asian J. Emp. Res..

[8] Thirtle C, Lin L, Piesse J (2003). The impact of research-led agricultural productivity growth on poverty reduction in Africa Asia and Latin America. World Dev..

[9] Ogundari K (2014). The Paradigm of agricultural efficiency and its implication on food security in Africa: what does meta-analysis reveal?. World Dev..

[10] Koirala KH, Mishra A, Mohanty S (2016). Impact of land ownership on productivity and efficiency of rice farmers: the case of the Philippines. Land Use Policy.

[11] Adeniyi DA, Dinbabo MF (2020). Efficiency, food security and differentiation in small-scale irrigation agriculture: evidence from North West Nigeria. Cogent Soc. Sci..

[12] Mujemdar S, Bala BK, Arshad FM, Haque MA, Hossain MA (2016). Food security through increasing technical efficiency and reducing postharvest losses of rice production systems in Bangladesh. Food Secur..

[13] Iheke OR, Onyendi CO (2017). Economic efficiency and food security status of rural farm households in Abia State of Nigeria. Am. J. Food Sci. Nutr..

[14] Oyakhilomen O, Daniel AI, Zibah RG (2015). Technical efficiency-food security nexus in Kaduna State Nigeria: a case study of poultry egg farmers. Cons. J. Sustain. Dev..

[15] Oyetunde-Usman Z, Olagunju KO (2019). Determinants of food security and technical efficiency among agricultural households in Nigeria. Economies.

[16] Abdullah D (2019). Factors affecting household food security in rural northern hinterland of Pakistan. J. Saudi Soc. Agric. Sci..

[17] Holden ST, Ghebru H (2016). Land tenure reforms, tenure security and food security in poor agrarian economies: causal linkages and research gaps. Glob. Food Sec..

[18] Mishra AK, Bairagi S, Velasco ML, Mohanty S (2018). Impact of access to capital and abiotic stress on production efficiency: evidence from rice farming in Cambodia. Land Use Policy.

[19] Emran S, Shilpi F (2018). Agricultural productivity, hired labor, wages, and poverty: evidence from Bangladesh. World Dev..

[20] Mellor JW, Malik SJ (2017). The impact of growth in small commercial farm productivity on rural poverty reduction. World Dev..

[21] Montaud JM, Pecastaing N, Tankari M (2017). Potential socio-economic implications of future climate change and variability for Nigerien agriculture: a country wide dynamic CGE-Microsimulation analysis. Econ. Model..

[22] Guo B (2011). Household assets and food security: Evidence from the survey of program dynamics. J. Fam. Econ..

[23] Rose D, Gundersen C, Oliveira V (1998). Socio-Economic Determinants of Food Insecurity in the United States.

[24] Frongillo EA, Olson CM, Rauschenbach BS, Kendall A (1997). Nutritional Consequences of Food Insecurity in a Rural New York State County.

[25] Chang Y, Chatterjee S, Kim J (2014). Household finance and food insecurity. J. Fam. Econ..

[26] Kidane H, Alemu ZG, Kundhlande G (2005). Causes of household food insecurity in Koredegaga peasant association, oromiya zone. Ethiopia. Agrekon..

[27] Ali A, Khan MA (2013). Livestock ownership in ensuring rural household food security in Pakistan. J. Animal Plant Sci..

[28] Loopstra R, Tarasuk V (2013). Severity of household food insecurity is sensitive to change in household income and employment status among low-income families. J. Nutr..

[29] Riely, F., Mock, N., Cogill, B., Bailey, L. & Kenefick, E. *Food Security Indicators and Framework for use in the Monitoring and Evaluation of Food Aid Programs*. (Nutrition Technical Assistance Project (FANTA), Washington, DC.1999).

[30] Akpan, E. O. Oil resource management and food insecurity in Nigeria. *European Report on Development (ERD) Conference in Accra, Ghana*. (2009).

[31] Owusu V, Abdulai A, Abdul-Rahman S (2011). Non-farm work and food security among farm households in Northern Ghana. Food Policy.

[32] Kassie M, Ndiritu SW, Stage J (2014). What determines gender inequality inhousehold food security in Kenya? Application of exogenous switching treatment regression. World Dev..

[33] Bogale A (2012). Vulnerability of smallholder rural households to food insecurity in Eastern Ethiopia. Food Secur..

[34] Mango N, Zamasiya B, Makate C, Nyikahadzoi K, Siziba S (2014). Factors influencing household food security among smallholder farmers in the Mudzi district of Zimbabwe. Dev. South. Afr..

[35] Asghar Z, Muhammad A (2013). Socio-economic determinants of household food insecurity in Pakistan. MPRA Paper.

[36] Bashir MK, Schilizzi S, Pandit R (2013). Impact of socio-economic characteristics of rural households on food security: the case of the Punjab, Pakistan. JAPS J. Animal Plant Sci..

[37] Gill, A. R. & Khan, R. E. A. Determinants of Food Security in Rural Areas of Pakistan. *SSRN Working Paper Series*. (2010).

[38] Mainuddin M, Kirby M (2015). National food security in Bangladesh to 2050. Food Secur..

[39] Frelat R (2016). Drivers of household food availability in sub-Saharan Africa based on big data from small farms. PNAS.

[40] Ridaura S (2018). Climate smart agriculture, farm household typologies and food securityAnex-anteassessment from Eastern India. Agric. Syst..

[41] Diethmer J, Abdulai A (2017). Does trade openness contribute to food security? A dynamic panel analysis. Food Policy.

[42] Muraoka R, Jin S, Jayne TS (2018). Land access, land rental and food security: evidence from Kenya. Land Use Policy.

[43] Swindale, A. & Bilinsky, P. *Household Dietary Diversity Score (HDDS) for Measurement of Household Food Access: Indicator Guide (v.2)*. (Washington, D.C.: FHI 360/FANTA. 2016)

